# Sex Worker Health Outcomes in High-Income Countries of Varied Regulatory Environments: A Systematic Review

**DOI:** 10.3390/ijerph18083956

**Published:** 2021-04-09

**Authors:** Jessica McCann, Gemma Crawford, Jonathan Hallett

**Affiliations:** 1 Curtin School of Population Health, Curtin University, Kent Street, Bentley, WA 6102, Australia; g.crawford@curtin.edu.au (G.C.); j.hallett@curtin.edu.au (J.H.); 2 Collaboration for Evidence, Research and Impact in Public Health, Curtin School of Population Health, Curtin University, Kent Street, Bentley, WA 6102, Australia

**Keywords:** sex work, regulation, legislation, public health

## Abstract

There is significant debate regarding the regulation of the sex industry, with a complex range of cultural, political and social factors influencing regulatory models which vary considerably between and within countries. This systematic review examined the available evidence on the relationship between different approaches to sex industry regulation in high-income countries, and associated effects on sex worker health status. Objectives included identification of sex worker health outcomes, including sexual health, substance use and experience of stigma and violence. A search was performed electronically in eight scholarly databases which yielded 95 articles which met the criteria for inclusion. Findings suggested that sex workers in legalised and decriminalized countries demonstrated greater health outcomes, including awareness of health conditions and risk factors.

## 1. Introduction

Sex workers are a priority population for public health [1] and there is growing support for occupational health and safety approaches to support sex worker health [2,3,4]. Sex workers may experience vulnerability for a number of health issues, including those related to mental health, sexual health, substance use and interpersonal violence [5]. A recent study found higher rates of alcohol use, illicit drug use, and experiences of violence amongst sex workers compared to the general population [5]. Similar outcomes have been noted in other research, with concerns raised for the human rights of sex workers in response to increasing rates of violence [6,7] alcohol [8,9] and drug use [10]. Health issues in this population are exacerbated by the experience of discrimination and stigma, leading to reduced health service seeking behaviour [11,12].

Regulatory models for sex work are varied; shaped by social, cultural and political influences [13,14,15]. Historically, criminalisation was the favoured regulatory model in many high-income countries [16]. More recently, societal perspectives towards the sex industry have shifted, resulting in regulation that is increasingly diverse within and between countries [17]. For example, in Australia, regulations differ by jurisdiction, and include partial criminalisation in WA, decriminalization in New South Wales (NSW), and legalisation with licensing regulation in Victoria (VIC) [5]. In comparison, sex work is decriminalized in New Zealand [18]. Sex work is illegal in all states in the United States of America (USA) except in certain counties in Nevada, where licensed brothels are permitted [19]. In contrast, sex work is legal in Denmark, however it is illegal for a third-party, such as a brothel, to profit from sex work [20].

Recent literature has demonstrated an association between legislation and sex worker health outcomes [21]. Public preference is increasingly growing in favour of decriminalization [6,8,14,22,23], and evidence continues to support the effectiveness of decriminalization as a regulatory model for improving sex worker health outcomes [4,5,18,22,24,25,26,27,28]. Findings to date have demonstrated the benefits of decriminalization at a study level [18,29] and multiple jurisdictions, including in Australia, are considering reform to sex industry legislation [30,31,32]. This systematic review aimed to identify and critically appraise global findings on sex worker health outcomes within high-income countries of differing regulatory environments.

## 2. Materials and Methods

The review was performed in accordance with the Preferred Reporting Items for Systematic Reviews and Meta-Analyses (PRISMA) criteria [33] and registered with the International Prospective Register of Systematic Reviews (PROSPERO), registration number: CRD42018109964.

### 2.1. Study Eligibility

This systematic review included available, English language, full-text, peer-reviewed primary research using quantitative (experimental and epidemiological) methods which were conducted in high-income countries and published between 1978 and 2019. High-income countries were defined by the World Bank as countries with a Gross National Income (GNI) per capita of USD 12,056 and above for the year 2017 [34]. The review included studies with a focus on adult sex workers (over 18 years) and excluded child prostitution and trafficking. The legal age of adulthood and consent to sell sex varies globally within and between countries and cultures [35]. While application of a universal demarcation (e.g., 18 years) has been critiqued for its ethnocentrism [36], for comparative and pragmatic purposes and to reduce the likelihood of children being included as part of study populations, the authors defined adulthood as per the United Nations Convention on the Rights of the Child [37]. Articles in which sex workers had been trafficked, or the consent to sex work was ambiguous, were excluded. Articles from 1978 onwards were included in the literature search given in part the lasting impacts upon sex industry legislation resulting from the emergence of the HIV epidemic in the late 1970′s [13,18]. Due to the complex nature of sex work, many articles were noted that involved vulnerable population groups such as people who use drugs, people experiencing homelessness, incarcerated individuals, transgender people, people experiencing mental health disorders and individuals who had experienced physical or sexual abuse. Such articles were excluded if the primary focus was not sex work, or if sex work was utilised as an outcome measure rather than as a population descriptor. This approach was chosen to ensure that clarity of the research population was maintained.

### 2.2. Information Sources

Peer reviewed articles related to sex worker health and sex industry legislation were obtained electronically from the following academic databases: PubMed, ProQuest, Scopus, Current Contents Connect and Ovid (including Medline, Embase, PsycINFO and Global Health). Databases were selected through consultation with the university librarian, covering a broad set of clinical, social science and public health literature.

### 2.3. Search Strategy and Study Selection

A combination of keyword and subject heading/MESH heading terms were identified that included varied terminology for the population group, high-income countries and quantitative outcomes. This was to ensure that articles related to health outcomes and legislation were encompassed in the search. The search terms are listed in Table 1.

**Figure 1:**
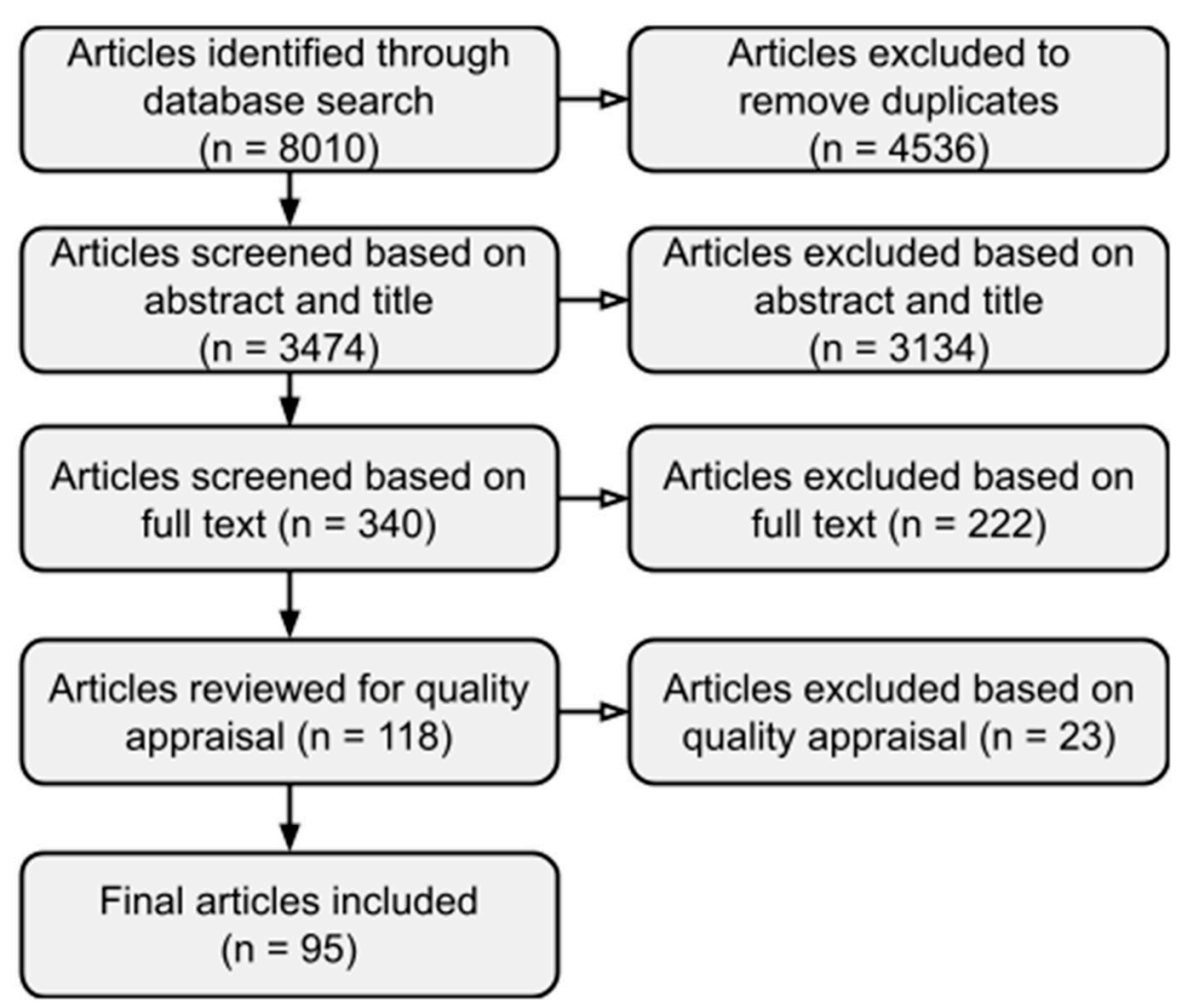
Database search strategy.

Terms were searched in the title and abstract of articles, with peer review selected as a filter for databases that supported this feature. Searches were conducted by the primary researcher (JM) and audited for consistency and accuracy by the other members of the research team Figure 1. presents the process undertaken for the review.

Articles were managed and stored using the citation management software, EndNote (version X8.2) (Clarivate Analytics, Philadelphia, PA, USA). Article titles and abstracts were reviewed to remove duplicate entries and to exclude clearly irrelevant articles from initial search results (e.g., those not related to sex work or reporting on qualitative findings). Article titles and abstracts were screened against the inclusion criteria by JM who met with the research team to discuss sample article including those which were unclear or ambiguous before proceeding to final review of full text articles against the inclusion criteria. Reasons for exclusion at full-text review included: unclear consent to sex work (*n* = 7), inability to extract data on high income countries (*n* = 10), sex workers aged 18 years and above (*n* = 130), and qualitative results only (*n* = 49) and availability of conference abstracts only (*n* = 26).

### 2.4. Data Extraction and Quality Appraisal

Joanna Briggs Institute (JBI) critical appraisal checklists were utilised to assess the quality of extracted articles [38]. Quality assessment included review of study methodology, design, execution and consideration of bias. On completion of the quality appraisal, 23 articles were excluded from the analysis. Reasons for exclusion at full text review were: unclear methodologies of exposure and/or evaluation (*n* = 10), limited sample size (*n* = 6), insufficiently matched cases and controls (*n* = 5) and significant lost to follow up rates (*n* = 1). Rationale for exclusion based on quality appraisal was reviewed and verified by a member of the research team experienced in systematic review methodology.

A standardised data extraction table was developed based on those used in previous reviews by the research team [39,40]. The following data were extracted: overview (authors, location, aim); study characteristics (study design, recruitment, sample size, response rate); participant characteristics (mean age, sex/gender, other); ethical approval; evaluation design and measures; and study findings.

For articles where legislation was not described, this information was sought from government sources. Articles that did not describe recruitment in standard terminology were assigned one of the following terms for analysis purposes: convenience sampling (assigned to articles that described recruitment at brothels, sexual health clinics or sex work venues); time-location sampling (assigned to articles that specified recruitment at a specific time and location); and snowball sampling (assigned to articles that stated recruitment by peers). Articles that used terminology such as “women” and “men” without specifying reference to either gender or biological sex have been interpreted as female and male gender, respectively.

Platt and colleagues [21] have proposed a typology of sex work legislative models which was broadly used to categorise studies in this review as follows:

- Full criminalization: Legislation whereby all aspects of sex work and sex work locations and/or establishments are prohibited.
- Partial criminalization: Organisation of sex work is prohibited (e.g., involvement of third parties or running a brothel).
- ‘Nordic model’: Criminalization of purchase of sex and third parties.
- Legal: Regulatory models whereby sex work and sex work locations and/or establishments are legal (e.g., using a licencing or registration model).
- Decriminalization: Legislation whereby sex work and sex work locations and/or establishments are decriminalized. Criminal law may remain surrounding safe sex practices.

## 3. Results

Articles were collated and synthesised based on emerging themes in the review. Ninety-five studies met the criteria for inclusion. Results were subsequently categorised into the following domains: study location; legislation; participant demographics; sampling; study design; and health outcomes.

### 3.1. Study Location

Included articles captured data from a range of high-income countries. Four or more studies were conducted in the following countries: Argentina [41,42,43,44,45,46], Australia [27,29,47,48,49,50,51,52,53,54,55,56,57,58,59,60,61,62,63,64], Canada [65,66,67,68,69], Hong Kong [70,71,72,73,74,75,76,77], Italy [78,79,80,81], Japan [82,83,84,85,86], Singapore [87,88,89,90], Spain [91,92,93,94,95,96,97,98,99], The Netherlands [100,101,102,103,104,105,106,107] and the USA [108,109,110,111,112,113,114,115]. Two studies were found in each of the following countries: England [116,117], New Zealand [118,119], Portugal [120,121] and Scotland [122,123]. One study was conducted in each of the following countries: Belgium [124], Chile [125], Czech Republic [126], Denmark [127], Estonia [128], Hungary [129], Panama [130], Puerto Rico [131], South Korea [132], and Switzerland [133]. Some studies were multi-jurisdictional for example Australian studies that compared three or more states [27,53,54,55,56].

### 3.2. Legislation

Sex work legislation varied across the studies, with the largest proportion of studies conducted in countries with partial criminalization [41,42,43,44,45,46,47,62,63,66,67,68,69,70,71,72,73,74,75,76,77,78,79,80,81,82,83,84,85,86,87,88,89,90,91,92,93,94,95,96,97,98,99,101,102,105,116,117,119,120,121,122,123,124,127,128]. A summary of the legal status of sex work activity in included studies is described in Table 2 [134]. A more comprehensive summary of included studies can be found in Supplementary Materials (Tables S1–S6) reported by legislative framework: criminalized (Table S1: *n* = 10); partially criminalized (Table S2: *n* = 56); Nordic model (Table S3: *n* = 1); legalized (Table S4: *n* = 18; decriminalized (Table S5: *n* = 5) and multi-jurisdictional with differing legal statuses (Table S6: *n* = 5).

The second largest group of studies were those which focused on locations where sex work was legal [48,49,51,52,59,60,61,64,100,103,104,106,107,125,126,129,130,133] followed by criminalized [108,109,110,111,112,113,114,115,131,132]. Five studies were included where sex work was decriminalized [29,50,57,58,118] and one study was conducted in a setting with the Nordic model [65]. Five studies included multiple jurisdictions with legal, criminalized and partially criminalized settings [27,53,54,55,56].

### 3.3. Participant Characteristics

The mean age of participants for all studies ranged from 23.0 to 39.3 years of age. Most studies featured only female participants [27,29,42,45,46,47,48,50,51,52,53,57,58,59,60,61,64,67,68,70,71,72,73,74,75,76,77,78,79,80,81,82,83,84,85,86,87,88,89,90,92,93,94,95,96,97,99,100,101,102,103,104,105,107,109,112,113,115,116,119,122,124,125,126,127,128,130,131,132,133]. Eight studies had all male participants [54,55,56,69,91,111,117,121], whilst a smaller proportion of studies featured both male and female participants [49,66,110,123,129]. Twelve studies included transgender participants [41,43,44,62,63,65,98,106,108,114,118,120], three of which focused solely on transgender sex workers [41,44,108]. The remainder included transgender participants in addition to cisgender male and/or female participants [43,62,63,65,98,106,114,118,120]. This review included all types of transactional sex, with commercial sex trading comprising the largest proportion of articles [27,29,41,42,43,44,45,46,47,48,49,50,51,52,53,54,55,56,57,58,59,60,61,62,63,64,65,66,67,68,70,71,72,73,74,76,78,79,80,81,82,83,84,85,86,87,88,89,90,91,92,93,94,95,96,97,98,99,100,101,102,103,104,105,108,109,110,111,112,113,114,116,117,118,119,120,121,122,123,124,125,126,127,128,129,130,132,133]. A smaller proportion of studies focused on opportunistic sex trading (such as survival sex and sex for drugs) [69,77,115,131]. For example, two studies focused on the role of unfavourable living conditions in opportunistic sex work [69,77].

### 3.4. Study Design

Convenience sampling was the most common participant recruitment method [27,29,41,42,46,47,48,49,50,51,54,55,56,58,60,61,64,65,70,71,72,74,75,76,78,79,80,82,83,84,85,86,88,90,91,92,93,94,96,97,99,100,101,102,103,104,105,106,108,110,111,112,115,116,117,118,120,121,126,127,129,131,132]. Some studies used other non-probability sampling methods, such as snowball sampling [43,44,62,63,69,77,89,119,123], purposive sampling [45,52,53,57,59,65,66,67,73,98,100,107,108,113,118,124] and time-location sampling [68,81,87,114,122,125,130]. Additional recruitment methods found by one study each include cluster sampling [109], convenience stratified sampling [95], non-proportional quota sampling [133], and respondent driven sampling [128].

The majority of studies reviewed were cross-sectional studies and prevalence studies [27,29,41,42,43,44,46,47,48,49,50,51,53,54,55,56,57,58,59,60,61,62,63,64,65,66,67,68,70,71,72,73,74,75,76,77,78,79,80,81,85,86,90,91,93,95,97,98,99,100,103,104,105,106,107,108,109,110,111,112,114,115,118,119,120,121,122,123,125,126,128,129,130,131,132,133]. There were a smaller number of case-control studies [82,83,84,94,116,117,127], cohort studies [69,89,92,96,101,102,124] and quasi-experimental studies [45,87,88] as well as one randomised intervention trial [113] evaluating a HIV behavioural intervention conducted in the USA with drug involved female sex workers (*n* = 597) and STI clinical audit [52] conducted in Melbourne, Australia with female sex workers operating in a regulated environment (*n* = 388).

Most studies collected data through the use of questionnaires [27,29,45,47,50,51,55,60,61,62,63,65,66,68,69,70,71,72,74,76,77,81,88,90,98,99,103,108,114,115,118,119,121,125,129,133]. Less frequently used data collection methods included structured interviews [100,113,123] and self-completed diaries reporting on sex practices [54] and drug and alcohol use [56]. Twenty four studies utilised a combination of clinical testing and questionnaire data [41,42,44,46,48,67,75,79,80,85,87,89,91,105,106,109,110,111,112,120,127,130,131,132]. Sixteen studies utilised clinical testing data alone [43,73,78,82,83,84,86,92,94,95,96,101,104,124,126,128]. Nine studies used epidemiological data [49,52,53,57,58,59,107,116,117]. Three studies included both clinical testing and structured interview data [93,97,122]. Epidemiological data were combined with self-report STI diagnosis data reported by questionnaire [102] and with clinical laboratory STI testing [64] for two studies.

Fifty-two studies confirmed the approval of their research by a Human Research Ethics Committee (HREC) [27,29,41,43,44,46,48,49,50,51,53,54,55,56,57,58,59,60,61,62,63,64,65,66,68,71,72,76,77,87,93,94,96,97,98,99,103,104,106,108,109,113,118,120,121,123,125,128,130,132,133]. Three articles stated that HREC approval was not required for their research objectives [52,107,129]. The remainder did not report on ethical approval.

### 3.5. Health Outcomes

Studies reported a range of health outcomes. Mental health issues were most frequently reported [29,41,42,43,44,45,46,48,49,50,52,53,54,56,57,58,59,60,61,64,65,66,67,68,69,70,71,72,73,74,75,76,77,78,79,80,81,82,83,84,85,86,88,89,90,91,92,93,94,95,96,97,98,99,100,101,102,103,104,105,106,107,108,109,110,111,112,115,116,117,120,121,122,123,124,126,127,128,129,130,131,133]. This included: higher distress levels compared to non-sex workers in the USA (*n* = 176) [115], social isolation reported by Asian sex workers in Western Australia (*n* = 94) [63] and high rates of mental health disorders experienced by sex workers in Switzerland (*n* = 193) [133]. Experience of violence [41,46,47,77,98,108,114,133], stigma [27,44,51,63,118], drug use [29,41,56,66,80,98,110,123,129,131], rates of sexually transmissible infections (STIs) and bloodborne viruses (BBVs) [41,42,43,44,48,49,52,53,57,61,64,67,69,73,75,78,79,80,82,83,84,85,86,88,89,91,92,93,94,95,96,97,100,101,102,104,105,106,107,109,110,111,112,113,116,117,120,121,122,123,124,126,127,128,130,132] and use of health services [27,29,45,47,48,55,65,71,72,76,77,102,113,116,117,119,125] were also reported. Two of the five studies that reported experiences of stigma as a health outcome also noted experience of stigma as a risk factor for reduced usage of health services by sex workers [27,118]. For example, the study by Abel found that sex workers frequently did not disclose their profession to health care providers due to fears of stigmatization, leading to less comprehensive health reviews [118].

Studies from settings where sex work was criminalized and partially criminalized frequently reported epidemiological data such as prevalence of STIs and BBVs, vaccination rates and drug use [78,79,80,82,83,84,85,86,109,111,112,122,123]. However, it was noted that epidemiological data for partially criminalized studies was more often reported in the context of social influences such as experience of violence [41,46], stigma [44] and sexual risk behaviours [66,67,69,88,89,101,105,121,127]. Studies in settings where sex work was legalized commonly reported on legislation effects upon the improvement of sexual health [52,61] and mental health outcomes [60,103]. Studies in settings where sex work was decriminalized showed sex workers were likely to engage in health service seeking behaviour [18,29,57]. These findings were contrasted by a number of studies which identified issues of access to and usage of health services in the other contexts including partially criminalized [45,72,116,117,119], criminalized [113,114] and “Nordic model” settings [65]. A study explicitly comparing health service access between decriminalized, legalized and partially criminalized jurisdictions in Australia found that sex workers in partially criminalized settings experienced the poorest health and safety outcomes, with greater availability of public sexual health clinics in legalized jurisdictions and most significant investment in health promotion programs and occupational health and safety measures in decriminalized and regulated settings [27,102].

Drug use was more frequently reported in studies from criminalized and partially criminalized settings [80,102,108,110,115,131]. One study from NSW in Australia (decriminalized), reported a reduction in drug use [29]. Studies from decriminalized and legalized locations mostly displayed consistent and improved condom usage [29,50,57,58,106], in comparison with studies from criminalized and partially criminalized settings that showed higher rates of poor condom usage [46,62,90,108,132]. The study from a Nordic model setting found higher prevalence of unmet health care needs, including poor mental health [65], Studies in partially criminalized settings noted both high STI prevalence [73,86,93,94,124] and low STI prevalence [79,97]. However, a study of multiple jurisdictions in Australia including legalized, decriminalized and partially criminalized settings, found significantly greater gonorrhea diagnoses in partially criminalized settings; attributed to increased policing of condom use [53].

## 4. Discussion

This review aimed to synthesize the available evidence on sex worker health in the context of different approaches to sex industry regulation in high-income countries. Ninety-five articles were identified for inclusion, published since 1978. Most studies were cross-sectional, using convenience sampling.

Cross-sectional design was common (*n* = 69) and a majority of articles used convenience sampling (*n* = 60) which may have led to measurement error [135]. The high proportion of self-report data and convenience sampling methods also introduces recall and self-selection bias [136,137]. Such methodologies however are an accepted method for use with hard-to-reach populations such as sex workers, where recruitment can be impacted by fears of stigma or incrimination [138]. A large proportion of studies (*n* = 59) failed to adequately describe recruitment methodology and instead listed the location/s where recruitment occurred. Further limitations include the lack of information on the use of standardised and validated questionnaires as an evaluation method. As such the reliability and validity of these data is unclear. Almost half the included studies did not report ethical approval (*n* = 40). Three quarters of these (*n* = 30) were published prior to the year 2006. This correlates with changes in reporting requirements, as increased transparency in reporting HREC approval has been mandated in recent times [139]. There is a call to action for high-quality, robust studies with ethical oversight given the inherent challenges relating to power and coercion particularly where legality intersects with experiences of marginalization.

Sex workers in legalized and decriminalized contexts demonstrated greater awareness of health conditions and health risk behaviours, in comparison with criminalized jurisdictions. Studies in criminalized settings reported a higher proportion of drug use [56,108,122,129] associated with depression [131] distress [115] and reduced condom use [108]. This review found that criminalization of sex work increased risk of poorer social and health outcomes [108,109,114,115,131]; a finding which is consistent with the existing literature [5,18]. The literature identifies concomitant factors such as homelessness [140] and incarceration [141] which may also have impacts upon sex worker health outcomes. While only one of the included studies in this review used the Nordic model, this approach has been criticized for spatial displacement of sex workers and increasing risk to sex workers in their negotiation with clients [142]. Given the relatively few empirical studies of the impact of the Nordic Model on workers in the literature [143] and its increasing popularity across many high-income countries, there is an urgent need for additional research to test underlying assumptions of the model that suggest it reduces sex work numbers and harms.

Greater condom usage was seen in legalized and decriminalized contexts. For example, the study by Van Veen et al. reported consistent condom use in 81% of participants [106]. Lower condom use in in some criminalized jurisdictions, may be in part influenced by the use of carrying condoms as evidence of criminal activity by law enforcement, creating barriers to carrying or using condoms [144,145]. This may also account for some of the higher STI prevalence rates reported in partially criminalized settings [67,69]. For example, studies in these settings found that sex workers experienced greater pressure from clients to have unprotected sex [67] and offers for higher payments to have condomless sex [66]. Levy and colleagues [142] also note low provision of condoms in Sweden via designated prostitution units, due in part to perception that provision of condoms facilitated sex work, which was inconsistent with the state aims of Sweden’s abolitionism.

For those studies published in the early 1990s, the impacts of the HIV epidemic on research practices throughout this time became evident. Studies from this decade were largely epidemiological, with a focus on HIV prevalence and the role of sex workers as a vector for transmission [97,101,105,110,111,122,123,131]. Community and political responses to the emerging Australian HIV epidemic resulted in significant law reforms within the sex industry, with changes in sex practices and policies emerging in this time [146]. From 1998 onwards, there was a shift away from an HIV focus in the included studies. This aligns with the availability of HIV treatments and subsequent changes in health priority areas as the burden of disease from HIV decreased in high-income countries [147]. Although this finding is not a reflection of the current laws, it does highlight the impacts of HIV upon high-income countries. Sex workers are a priority population group for HIV prevention strategies for both higher and lower income countries [46,67,79,87,106]. It is therefore of value to understand how social and environmental characteristics of sex work and sex work legislation have been shaped from the historical and current context of HIV prevention strategies and policies. There is great need and opportunity for future research to include sex workers in peer-based roles. Findings suggest that peer researchers facilitate sex worker support and increase research impacts [148]. For example, a study by Selvey et al. including peer-based researchers found that peer outreach and support was beneficial for improvement of sexual health and education, particularly for sex workers from culturally and linguistically diverse backgrounds [63].

Sex workers may have greater accessibility to health promotion programs including access to free and anonymous testing [27] and other health services in locations where brothels are not criminalized. Research from NSW has found greater usage of health promotion programs by sex workers in decriminalized cities [27]. Other authors have observed high rates of voluntary sexual health checks in decriminalized jurisdictions and suggest that mandatory testing schemes have not been a feature of successful intervention strategies despite political saliency [149]. Some studies in criminalized and partially criminalized jurisdictions also showed the effectiveness of health promotion programs for improving use of health services [45,113], and reducing health risk behaviours [87,88]. The sort of program highlighted as effective were peer to peer education [27] and sexual health education programs focused on STI prevention and condom usage [42,88,100]. These findings highlight the critical role that health promotion programs should and do play in health education and awareness among sex workers in countries where sex work is currently criminalized. Despite stated need amongst participants, research by Levy and colleagues highlighted poor harm reduction coverage and conditionality in service provision within the Swedish context [142]. Investment in resources and funding for sex work advocacy groups and non-governmental organisations is needed to ensure the continuation of valuable health promotion programs regardless of legal context.

This review found experiences of stigma, discrimination and marginalization across countries of varying legislation, with experience of stigma associated with reduced use of health services. For example, findings from NSW indicate an improvement in the health and safety of the sex industry, in addition to reduced financial burden on the criminal justice system, since introduction of decriminalization in 1995 [22]. It is also argued that decriminalization has the potential to normalize the role of sex work, thereby reducing the stigma and discrimination experienced amongst sex workers [26] and increasing the accessibility of health services [5]. Previous studies have suggested that experience of social issues such as stigma, marginalization and discrimination may remain for some time post legislative reform [18,24]. This could account for presence of stigma in countries with decriminalization and legalization, where systemic and historic marginalization has impacted upon access to and use of health services [103,118,119]. This finding suggests that sex workers’ experience of discrimination and stigma may take time to improve post decriminalization. Established cultural norms, including societal attitudes and behaviours towards sex work can be slow to change, particularly given the historical context of criminalisation [13]. This highlights the pervasive nature of stigma [150] which requires further investigation through more robust study designs that include analysis of the political, social and cultural factors that shape experience of stigma and discrimination. Specific interventions and policies in addition to legislation are required to improve this social health outcome.

This review had a number of strengths and limitations. The review provides a comprehensive 40-year picture of the literature. The use of eight databases provided expanded scope. To increase rigour, the review followed an established protocol registered with the PROSPERO International Prospective Register of Systematic Reviews. The review was restricted to articles published in English. Inclusion of papers in languages other than English may have yielded relevant information and a broader range of data. Given the heterogeneity in culture and health care systems among high-income countries making comparisons should be approached with caution.

We recognize that important information may be located in the non-peer reviewed literature including from authors who do not have the resources to publish findings in peer reviewed journals. The lack of inclusion of grey literature may have led to publication bias. Inclusion criteria review was only performed by one researcher, however a small sub-section of articles was reviewed by the research team, to assist with standardisation of the review process. The breadth of inclusion criteria increased the heterogeneity of results, consequently no meta-analysis was conducted.

Finally, it is noted that use of search terms “sex work/ers” may increase selection bias towards articles in which individuals identify with this term as a profession. It is understood that not all individuals who engage in transactional sex consider themselves to be a sex worker. Although inclusion criteria did not specify type of transactional sex, it is considered that the larger volume of articles pertaining to commercial sex work is explained by the search terminology utilised. It is possible that individuals who identify with sex work as a profession may have better health outcomes and differing socioeconomic factors than those who do not, although more research is required to support this suggestion.

## 5. Conclusions

Sex work laws are highly variable at a global and national level, with regulation influenced by political, social and cultural factors. There is growing evidence to support decriminalization as an approach to improve sex worker health and safety. Findings from this review highlight that criminalization of sex work increased risk of poorer social and health outcomes. Experiences of stigma, discrimination and marginalization is seen across countries of varying legislation and is associated with reduced use of health services. The review provides insights into the health and legal status of sex workers in high-income countries and calls for action to improve research design, address stigma and discrimination, and improve health education delivery.

## Figures and Tables

**Table 1:** Database search terminology.

Database	Subject Headings/MESH Terms	Keywords
PubMed	Sex work Sex workers Developed countries	(1) (prostitut* OR “sex work*” OR “sex industry” OR “sexual service” OR escort OR brothel OR “sex trade*”) (2) results AND (mean OR median OR outcome OR “standard error” OR “standard deviation” OR “odds ratio” OR prevalence OR cohort OR cross-section OR “cross section” OR “case control” OR prospective OR retrospective OR trial OR size OR quant* OR amount OR number OR survey* OR questionnaire) (3) (Andorra or “Antigua and Barbuda” or Argentin* or Aruba or Australia* or Austria* or Bahamas or Bahrain* or Barbados or bilge* or Bermuda or “British Virgin Island*” or Brunei or Canad* or “Cayman Island*” or “Channel Island*” or Chile* or Croatia* or Curaçao or Cyprus or “Czech Republic” or Denmark or Estonia or “Faroe Island*” or Finland or France or “French Polynesia” or Germany or Gibraltar or “Great Britain” or Greece or Greenland or Guam or “Hong Kong” or Hungar* or Iceland* or Ireland or “Isle of Man” or Israel* or Ital* or Japan* or Korea* or Kuwait or Latvia* or Liechtenstein or Lithuania or Luxembourg* or Macao or Malta or Monaco or Netherlands or “New Caledonia” or “New Zealand” or “Northern Mariana Island*” or Norw* or Oman or Palau or Panama or Poland or Portug* or “Puerto Ric*” or Qatar or “San Marino” or “Saudi Arabia*” or Scotland or Seychelles or Singapore* or “Sint marten” or “Slovak Republic” or Slovenia or Spain or “Saint Kitts and Nevis” or “Saint Martin” or Swed* or Switzerland or Taiwan* or Trinidad or Tobago or “Turks and Caicos” or “United Arab Emirates” or “United Kingdom” or “United States” or Uruguay or “Virgin Island*” or Wales)
ProQuest		
Scopus		
Current Contents Connect		
Medline	Sex work Sex workers Developed countries	
Embase	Sex worker Prostitution Developed country	
PsycINFO	Prostitution Developed countries	
Global Health	Sex workers Prostitutes Prostitution Developed countries	

**Table 2:** Summary of sex work legal status by study location and year of data collection.

Legal Status	Location of Included Studies (n)
Criminalized	Puerto Rico (*n* = 1); South Korea (*n* = 1); USA (*n* = 8)
Partial criminalization	Argentina (*n* = 6); Australia, WA (*n* = 3); Australia, QLD (prior to 1999) (*n* = 1); Australia, SA (*n* = 1); Belgium (*n* = 1); Canada (prior to 2014) (*n* = 4); Denmark (*n* = 1); England (*n* = 2); Estonia (*n* = 1); Hong Kong (*n* = 8); Italy (*n* = 4); Japan (*n* = 5); Netherlands (prior to 2000) (*n* = 3); New Zealand (prior to 2003) (*n* = 1); Portugal (*n* = 2); Scotland (*n* = 2); Singapore (*n* = 4); Spain (*n* = 9)
Nordic	Canada (from 2014) (*n* = 1)
Legal	Australia, QLD (from 1999) (*n* = 5); Australia, VIC (*n* = 11); Chile (*n* = 1); Czech Republic (*n* = 1); Hungary (*n* = 1); Netherlands (from 2000) (*n* = 5); Panama (*n* = 1); Switzerland (*n* = 1)
Decriminalization	Australia, NSW (*n* = 9); New Zealand (from 2003) (*n* = 1)

Note: Not included here is indirect criminalization of migrant sex workers who face additional challenges relating to citizenship in otherwise legal or decriminalized settings (see for example Selvey and colleagues [63]). Some studies included multiple jurisdictions and therefore appear in this table in multiple categories.

## Data Availability

The data presented in this study are available in Supplementary Materials Tables S1–S6.

## Supplementary Materials

The following are available online at https://www.mdpi.com/article/10.3390/ijerph18083956/s1, Table S1: summary of studies in criminalized jurisdictions (*n* = 10), Table S2: summary of studies in partially criminalized jurisdictions (*n* = 56), Table S3: summary of studies in ‘Nordic model’ jurisdictions (*n* = 1), Table S4: summary of studies in legalized jurisdictions (*n* = 18), Table S5: summary of studies in decriminalized jurisdictions (*n* = 5), Table S6: summary of studies involving multiple jurisdictions with different regulatory approaches (*n* = 5).
