# Supplementary material for: Sex Worker Health Outcomes in High-Income Countries of Varied Regulatory Environments: A Systematic Review
Source: Int J Environ Res Public Health. 2021 Apr 9;18(8):3956. doi: 10.3390/ijerph18083956 (PMC8070506; doi:10.3390/ijerph18083956)
Supplement: Supplementary file 1 [file ijerph-18-03956-s001.pdf]

Table: S1. Summary of studies in criminalized jurisdictions (n=10)

**Study**<br>**Location**<br>**Aim**	**Study design**<br>**Recruitment**<br>**Sample (n)**<br>**Response Rate (RR)**	**Participant characteristics:**<br>**Mean age**<br>**Gender**	**Ethical approval**	**Evaluation design and measures**	**Study findings**
Alegria et al. (1994).<br>*Puerto Rico.*<br>Association between depressive symptoms, HIV infection and risk behaviours	Cross-sectional<br>Convenience<br>n=127	32 years<br>Female	Not specified	HIV and syphilis testing. Self-report of depressive symptoms via CESDS questionnaire.	IDU and unprotected sex associated with high level of depressive symptoms. 70% had high levels of depressive symptoms. SWs with depressive symptoms more likely to report risk behaviours for HIV.
Lee et al. (2010).<br>*South Korea.*<br>To evaluate condom use and prevalence of chlamydia	Cross-sectional<br>Convenience<br>n=999<br>RR=92%	Age not specified<br>Female	Yes	STI testing. Condom use and sexual behaviours via questionnaire.	Condom use was low and corresponded to high prevalence of chlamydia.
Clements-Nolle et al. (2008).<br>*USA, California, San Francisco.*<br>To assess psychosocial correlates of condom use within this population	Cross-sectional<br>Purposive<br>n=190	31.5 years<br>Transgender	Yes	Condom use, health history and sexual practices via self-report questionnaire.	20% of participants reported inconsistent condom use.

Cohan et al. (2005).<br>*USA, California.*<br>To characterise demographics, sexual and drug using behaviours and prevalence of HIV, STI, and HAV, HBV, and HCV in low-income women with/without a history of SW	Cross-sectional<br>Cluster<br>n=226<br>RR=71.50%	26 years<br>Female	Yes	STI and HIV testing. Demographics, sexual behaviour and drug use via questionnaire	Women with a history of SW more likely to have positive results for syphilis, HSV-2 and HCV.
El-Bassel et al. (1997).<br>*USA, New York City, Harlem.*<br>To determine the relationship between sex trading and psychological distress	Cross-sectional<br>Convenience<br>n=176	26 years<br>Female	Not specified	Psychological distress via BSI questionnaire	SWs had higher levels of distress compared to non SWs. Drug use and mental health issues associated with higher distress levels.
Jones et al. (1998).<br>*USA.*<br>To examine prevalence of sexual risk behaviours and HIV and STI	Cross-sectional<br>Convenience<br>n=419	Age not specified<br>Female, Male	Not specified	STI and HIV testing. Assessment of sexual risk behaviours by questionnaire.	25% of participants infected with HIV, 37.5% with syphilis. Illicit drug using SWs had higher prevalence of risky sexual behaviours.
Morse et al. (1991).<br>*USA, Louisiana, New Orleans.*<br>To determine HIV prevalence and transmission	Prevalence<br>Convenience<br>n=211<br>RR=97%	Age not specified<br>Male	Not specified	STI testing. SW characteristics and risk behaviours via questionnaire.	HIV seroprevalence higher compared to FSW data from the CDC. MSW serve as a vector for HIV transmission.
Rosenblum et al. (1992).<br>*USA.*<br>To determine prevalence of HBV and evaluate transmission of HBV	Cross sectional<br>Convenience<br>n=1368	28 years<br>Female	Not specified	STI testing. Health history and STI diagnosis status via questionnaire.	Prevalence of past or present HBV infection was 56%.

Surratt et al. (2014).<br>*USA, Miami.*<br>To determine use of a behavioural intervention to reduce HIV risk in drug-using FSW	Randomized intervention trial<br>Purposive<br>n=597	39.3 years<br>Female<br>Participants from 14 Italian cities	Yes	Heath service use and perceived HIV risk pre/post intervention via computer assisted interviews	Increases in health service utilization and reduction in HIV risk
Valera et al. (2001).<br>*USA, Washington DC.*<br>To determine health needs of inner-city SWs	Cross-sectional<br>Time-location<br>n=100<br>RR=71.4%	30.61 years<br>Female, Male, Transgender	Not specified	SW characteristics and health issues via questionnaire	Major health needs include protection from assault, counselling, medical care, job training, social support.

Table: S2. Summary of studies in partially criminalized jurisdictions (n=56)

**Study**<br>**Location**<br>**Aim**	**Study design**<br>**Recruitment**<br>**Sample (n)**<br>**Response Rate (RR)**	**Participant characteristics:**<br>**Mean age**<br>**Gender**	**Ethical approval**	**Evaluation design and measures**	**Study findings**
Avila et al. (2017).<br>*Argentina.*<br>To determine the association of socio-demographic characteristics with syphilis and HIV infection.	Cross-sectional<br>Convenience<br>n=273	Not specified<br>Transgender	Yes	STI via blood sampling. Socio-demographic and health information, time as a SW, condom use in the last 6 months and sexual history via questionnaire.	Violence and substance use decreased SW ability to negotiate condom use and led to increased risk for transmission of HIV and STIs.
Bautista et al. (2009).<br>*Argentina.*<br>To determine socio-demographics, sexual practices, drug use behaviours, and prevalence of HIV, syphilis, HBV, HCV, HTLV-1 and 2 in migrant and non-migrant female SWs	Cross-sectional<br>Convenience<br>n=625	Age not specified<br>Female	Not specified	HIV and STI via blood sampling. Risk factors associated with migrant status via questionnaire.	Prevalence of syphilis and HCV significantly higher among Argentinean FSW than migrant FSW. HBV prevalence higher among migrant FSW.
Dos Ramos et al. (2011a).<br>*Argentina.*<br>To assess incidence of HIV and prevalence of HIV, HBV, HCV and HPV	Prevalence/Cross-sectional<br>Snowball<br>n=387<br>RR=90%	29 years (TSW)<br>27 years (MSW)	Yes	HIV, HBV, HCV and HPV via blood sampling	High incidence of HIV and prevalence of STIs.

Dos Ramos et al. (2011b).<br>*Argentina, Buenos Aires.*<br>To determine infecting HPV genotypes in anal cells and association between HPV infecting genotypes and HIV serological status.	Prevalence/Cross-sectional<br>Snowball<br>n=119	30 years<br>Transgender	Yes	HPV genotypes via blood sampling. Demographics via questionnaire.	High HPV prevalence, greater frequency of high-risk genotypes and marginalization and stigma.
Marin et al. (2015)<br>*Argentina, Buenos Aires, La Plata.*<br>To propose a health care model for SWs	Quasi-experimental<br>Purposive<br>n=840	30.2 years<br>Female	Not specified	Health care service use and risk behaviours via questionnaire	Baseline: 90.1% lacked health care access; 99.7% lacked health insurance. Post intervention: risky sexual behaviours decreased by 25%; health services access increased significantly.
Pando et al. (2013).<br>*Argentina.*<br>To assess frequency and type of violence experiences and the association with condom use and HIV prevalence	Cross-sectional<br>Convenience<br>n=1255	33.5 years<br>Female	Yes	HIV via blood sampling. Experience of violence, condom use and demographics via questionnaire	70% experienced at least one form of violence. Higher frequency of inconsistent condom usage amongst SWs who had experienced violence, sexual abuse, rejection and detection by police. Participants who had been arrested more likely to be HIV+.
Banach (1999).<br>*Australia, Queensland.*<br>To examine demographics, perception of danger of the sex industry, SW OHS, incidents of violence and sexual assault.	Cross-sectional<br>Convenience<br>n=149	Age not specified<br>Female	Not specified	Perceived danger and knowledge of SW law via questionnaire	Violence against SWs increasing and OHS decreasing. Current laws failed to protect SW OHS.

Selvey et al. (2018a).<br>*Australia, Western Australia.*<br>To investigate use of condoms by SWs during penetrative sex with clients	Cross-sectional<br>Snowball<br>n=354	Age not specified<br>Male, Female, Genderqueer	Yes	Rates and factors associated with condom usage via questionnaire	Increase in reporting condomless sex affected by increase in client demand, due to greater private SW and economic downturn.
Selvey et al. (2018b).<br>*Australia, Western Australia.*<br>To assess health and safety of Asian SWs	Cross sectional<br>Snowball<br>n=94	Age not specified<br>Male, Female, Genderqueer	Yes	Demographics and social characteristics of SW via questionnaire.	Asian SWs reported stigma, discrimination, social isolation, confusion about legalities of work and fear of police.
Mak et al. (2005).<br>*Belgium, Ghent.*<br>To determine prevalence of chlamydia	Cohort<br>Purposive<br>n=950	Age not specified<br>Female	Not specified	Urine testing	Chlamydia prevalence higher in SWs than general population.
Johnston et al. (2010).<br>*Canada, Vancouver.*<br>To examine prevalence and factors associated with being offered money for unprotected sex	Cross-sectional<br>Purposive<br>n=232	27 years,<br>Female, Male	Yes	SW practices and characteristics via questionnaire.	73.7% reported being offered more money to not use condoms; 30.6% accepted. Drug use associated with unprotected sex.
Shannon et al. (2007).<br>*Canada, Vancouver.*<br>To determine HIV prevalence and assess sexual and drug use related risks	Prevalence<br>Purposive<br>n=198	37 years<br>Female	Yes	HIV via blood sample. Demographics, drug use and health risk behaviours via questionnaire.	HIV prevalence 26%. SW Initiation at a younger age was associated with double the risk of baseline HIV+.

Shannon et al. (2009).<br>*Canada, Vancouver.*<br>To investigate relationship between condom use negotiation and environmental and structural factors	Prevalence<br>Time-location<br>n=205<br>RR=93%	37 years<br>Female	Yes	Characteristics of condom usage via questionnaire.	Pressure to have unprotected sex was associated with the location of work, affected by zoning restrictions and staying off main streets due to policing.
Weber et al. (2001).<br>*Canada, Vancouver.*<br>To determine the risk factors associated with trading sex	Cohort<br>Snowball<br>n=126 (SW)<br>n=761 (GP)	23 years (SW)<br>26 years (control)<br>Male	Not specified	Self-reported HIV diagnosis and risk factors via questionnaire	HIV incidence significantly higher amongst SWs than GP. HIV infection related to unfavourable living conditions, substance use, sexual risk behaviour.
Kjaer et al. (2000).<br>*Denmark, Copenhagen.*<br>To examine prevalence and risk factors for HPV in FSW compared to population	Case Control<br>Convenience<br>n=188 (FSW)<br>n=187 (controls)	32.1 years<br>Female	Not specified	HPV testing. Risk factors via questionnaire.	HPV prevalence declined with SW increasing age. Client numbers associated with risk of HPV infection, with a protective effect of condom use.
Grath-Lone et al. (2014a).<br>*England, London.*<br>To compare sexual health differences between FSWs and female population	Case control<br>Convenience<br>n=2704	28.5 years<br>Female	Not specified	Health clinics data demographics, STI prevalence, service use.	STIs more prevalent amongst SWs compared to general population. FSW had access to high quality care.
Grath-Lone et al. (2014b).<br>*England, London.*<br>To compare sexual health differences between MSWs and male population	Case control<br>Convenience<br>n=488	29 years<br>Male	Not specified	Health clinics data demographics, STI prevalence, service use.	Some STIs more prevalent in MSW than general population. Low uptake of health services by MSW.

Uuskula et al. (2008).<br>*Estonia, Tallinn.*<br>To determine prevalence of HCV and HIV	Cross-sectional<br>Respondent-driven<br>n=227	29.5 years, Female	Yes	STI testing blood test	HIV prevalence highest amongst street SWs.
Chan et al. (2002).<br>*Hong Kong.*<br>To identify epidemiological and behavioural characteristics of FSWs	Cross-sectional<br>Convenience<br>n=1451<br>RR=92.9%	Median range = 30-39 years<br>Female	Not specified	SW characteristics, risk behaviours and demographics via questionnaire	Majority of SWs of Chinese ethnicity. Increasing condom use by SWs.
Cheng et al. (2010).<br>*Hong Kong.*<br>To examine effects of NGOs, FSWs' managers, and clients on FSWs' negotiation efficacy and condom use efficacy, and the effects of efficacy on condom use.	Cross-sectional<br>Convenience<br>n=185<br>RR=64.4%	31.21 years<br>Female	Yes	HIV/STI knowledge and influence of managers, clients and NGOs on knowledge of safe sex practices via questionnaire	NGO influence and client support were positively related to negotiation self-efficacy. Manager and client pressure were negatively related to negotiation self-efficacy and condom use. SWs with high condom-use self-efficacy were 24 times more likely to use condom in the previous six months than counterparts.
Holroyd et al. (2008).<br>*Hong Kong.*<br>To assess effect of SW on women's environmental health and safety	Cross-sectional<br>Snowball<br>n=89	36.07 years<br>Female	Yes	Health and safety via validated quality of life questionnaire	Many SWs lived in sub-optimal conditions and risked abuse at work. SWs had lower environmental health scores than general population.

Lau et al. (2010).<br>*Hong Kong.*<br>To investigate prevalence of psychological problems and their relationships with HIV prevention behaviours	Cross-sectional<br>Convenience<br>n=293, RR=80%	Age not specified	Yes	Mental health and SW behaviours via quality-of-life questionnaire	Psychological problems associated with inconsistent condom use and low usage of prevention services.
Leung et al. (2013).<br>*Hong Kong.*<br>To investigate prevalence of pre-cancerous uterine cervical lesions	Prevalence<br>Purposive<br>n=2697	37 years<br>Female	Not specified	Rates of pre-cancerous uterine lesions via cervical pap-smear testing	Significantly higher prevalence of abnormal pap smears in SWs compared to the general population.
Ling et al. (2004).<br>*Hong Kong.*<br>To examine work environment factors associated with suicide	Cross-sectional<br>Convenience<br>n=89	36 years<br>Female	Not specified	Mental health and SW environment characteristics via questionnaire	Factors associated with sex industry correlated with poor psychological health and suicidality. One quarter had considered or attempted suicide.
Wong et al. (2006).<br>*Hong Kong.*<br>To investigate physical and psychological well-being of street FSW	Cross-sectional<br>Convenience<br>n=89	36.1 years<br>Female	Yes	Wellbeing and psychological factors associated with SW via validated quality of life questionnaire.	FSW scored low on quality of life. Social factors such as self-esteem, healthcare and feeling trapped affected SW health.
Wong et al. (2015).<br>*Hong Kong.*<br>To investigate prevalence of chlamydia and gonorrhoea	Cross-sectional, Convenience, n=340, RR=41%	37 years, Female	Not specified	HIV/STI blood testing and demographics via questionnaire	Prevalence of HIV and syphilis low. Prevalence of gonorrhoea high.

D'Antuono et al. (2001).<br>*Italy, Bologna.*<br>Prevalence of HIV and STIs	Prevalence/Cross-sectional<br>Convenience<br>n=558	23.5 years<br>Female,	Not specified	STI testing	SWs had no significant role in transmission of STIs. HIV prevalence increasing.
Nigro et al. (2006).<br>*Italy, Catania.*<br>To estimate prevalence of HIV, HBV, HCV and syphilis	Prevalence<br>Convenience,<br>n=119<br>RR=63.78%	38.3 years<br>Female	Not specified	STI testing. Condom usage behaviours via questionnaire.	Prevalence of HIV, HBV, HCV and syphilis low. Condom usage high.
Spina et al. (1998).<br>*Italy.*<br>To investigate condom use and HIV seroprevalence	Cross sectional<br>Convenience<br>n=802	37 years<br>Female	Not specified	HIV testing. Condom usage behaviours via questionnaire.	Increases in HIV seroprevalence amongst SWs. HIV seroprevalence amongst IDU SWs remains constant.
Trani et al. (2006).<br>*Italy, Milan.*<br>To assess sexual behaviour and STI knowledge	Cross-sectional<br>Time-location<br>n=241<br>RR=83.1%	29.8 years<br>Female	Not specified	Sexual health knowledge and behaviours via questionnaire	Knowledge of AIDS and syphilis common, knowledge of other STIs low.
Ishi et al. (2000a).<br>*Japan, Tokyo.*<br>To investigate prevalence of HPV, chlamydia and gonorrhoeae among SWs	Case control<br>Convenience<br>n=546 SW<br>n=233 controls	28.9 years<br>Female	Not specified	STI testing for HPV, CT and NG	High prevalence of HPV, CT and NG infection found in CSW.

Ishi et al. (2000b).<br>*Japan, Tokyo.*<br>To investigate the prevalence of HPV and association with cervical lesions among SWs	Case control, Convenience<br>n=546 SW<br>n=233 controls	28.9 years<br>Female	Not specified	HPV testing	High occurrence of HPV infection and cervical dysplasia were found in SWs.
Ishi et al. (2001).<br>*Japan, Tokyo.*<br>To assess prevalence of antibodies to HIV, HBV, HCV and HBs antigen in SWs	Case control<br>Convenience<br>n=308 SW,<br>n=384 control	28.2 years<br>Female	Not specified	STI and HIV testing.	Higher HCV antibody positive rate found in SWs.
Tanaka et al. (1998).<br>*Japan, Fukuoka.*<br>To investigate trend in STI and condom use patterns	Prevalence<br>Convenience<br>n=1218	28.3 years<br>Female	Not specified	STI testing. Condom use via questionnaire.	Prevalence of chlamydia, gonorrhoea and syphilis decreased significantly between 1990-1993 which may be related to increased condom use.
Tsunoe et al. (2000).<br>*Japan.*<br>To determine prevalence of chlamydia	Cross-sectional<br>Convenience<br>n=174	29.4 years<br>Female	Not specified	STI testing.	High prevalence of chlamydia, gonorrhoea, mycoplasma genitalium
Fennema et al. (1995a).<br>*The Netherlands, Amsterdam.*<br>To evaluate incidence of STIs in HIV positive SWs	Cohort study<br>Convenience<br>n=212	27 years<br>Female	Not specified	Rates of STI	PLHIV SWs had increased STIs and gynaecological disorders.

Fennema et al. (1995b).<br>*The Netherlands, Amsterdam.*<br>To determine occurrence of over and under self -reporting of STIs and test validity of self-reporting	Cohort<br>Convenience<br>n=131	28.2 years<br>Female	Not specified	Rates of STI diagnosis as per health record data compared to self-report data via questionnaire	34% of STI diagnosis were not reported. Misclassification of self-reporting is common. Self-reporting is unreliable.
Van Haastrecht et al. (1993).<br>*The Netherlands, Amsterdam.*<br>To determine HIV prevalence and sexual risk behaviour	Cross-sectional<br>Convenience<br>n=201<br>RR= 77% (clinic), 79% (non-clinic)	29 years<br>Female	Not specified	HIV testing, Consistency of condom use with clients in the previous 6 months via questionnaire	Low HIV prevalence. Consistent condom use reported by 66% of participants. All HIV cases were amongst recently arrived migrants.
Plumridge and Abel (2000).<br>*New Zealand, Christchurch.*<br>To examine the use of health services by FSWs	Cross-sectional<br>Snowball<br>n=303	Age not specified<br>Female	Not specified	Access to and use of health services via questionnaire	Majority of participants accessed a General Practitioner, however only half disclosed occupation of SW.
Dias et al. (2015).<br>*Portugal.*<br>To assess risk-taking behaviours and HIV prevalence	Cross-sectional<br>Convenience<br>n=1040<br>RR=65%	Age not specified<br>Female, Transgender	Yes	HIV via blood sample. HIV risk behaviours and testing behaviours via questionnaire.	HIV infection high in SWs.
Pereira et al. (2014).<br>*Portugal, Lisbon.*<br>To determine HIV prevalence and risk behaviours	Cross-sectional<br>Convenience<br>n=143	28.26 years<br>Male	Yes	Assessment of sexual risk behaviours, HIV diagnosis and demographics via questionnaire	Rates of condom use higher with clients than with partners. Risks included inconsistent HIV testing and limited perceived health risk.

Plant et al. (1990).<br>*Scotland, Edinburgh.*<br>To determine prevalence of alcohol use and AIDS risk	Cross sectional<br>Snowball<br>n=103 FSW, n=102 MSW	26 years (Female)<br>23 years (Male)	Yes	Structured interview to capture health status, alcohol and illicit drug use behaviours	Alcohol and illicit drug use common. 8.3% HIV positive.
McKeganey et al. (1992).<br>*Scotland, Glasgow.*<br>To identify extent of HIV infection and IDU amongst female street working SWs	Cross-sectional<br>Time-location<br>n=206	Age not specified, Female	Not specified	HIV testing. Structured interview to determine risk behaviours.	HIV infection rates not as prevalent as thought based on media reports (2-5% of samples).
Lim et al. (2018).<br>*Singapore.*<br>To assess efficacy of a HIV/STI prevention program on improving condom usage	Quasi-experimental<br>Time-location<br>n=220	31.5 years<br>Female	Yes	STI testing. Evaluation of program via condom usage questionnaire	Intervention group more likely to report consistent condom use than control group.
Wong et al. (1999).<br>*Singapore.*<br>To determine risk of contracting pharyngeal gonorrhoea by inconsistent condom usage	Cohort<br>Snowball<br>n=724	24.8 years<br>Female	Not specified	Rates of STI determined by throat swab. Condom usage via questionnaire.	SWs with inconsistent condom use were 17.1 times more likely to contract pharyngeal gonorrhoea.
Wong et al. (2000).<br>*Singapore.*<br>To determine prevalence of condom use and factors associated with consistent condom use	Cross-sectional<br>Convenience<br>n=225	28.5 years<br>Female	Not specified	Condom usage behaviours via questionnaire.	Half participants used condoms consistently for oral sex. 97% used condoms for vaginal sex. Negotiation skills and middle class associated with higher condom use.

Wong et al. (2004).<br>*Singapore.*<br>To evaluate long term impact of a condom promotion programme	Quasi-experimental<br>Convenience<br>n=273	25.1 years<br>Female	Not specified	Self-report consistent condom use via questionnaire.	Improved and sustained a high level of condom use for vaginal and oral sex. A decline in pharyngeal and cervical gonorrhoea incidence.
Belza (2005).<br>*Spain.*<br>To determine prevalence and risk factors for HIV	Cross-sectional<br>Convenience<br>n=418	29.2 years<br>Male	Not specified	HIV testing. Drug use, sexual practices and demography via questionnaire.	MSW less frequent than FSW although MSWs have higher risk for HIV infection and higher HIV prevalence.
Bratos et al. (1993.<br>*Spain.*<br>To examine influence of syphilis on HBV transmission, age and time as SW on HBV infection	Cohort<br>Convenience<br>n=368	Age not specified<br>Female	Not specified	STI testing. 6 monthly follow up for 2-4 years to assess SW characteristics via blood sampling	Age and length of time as SW increased with prevalence of HBV infection.
del Amo et al. (2009).<br>*Spain, Alicante.*<br>To examine differences by region of origin on prevalence and risk factors for high risk HPV	Cross-sectional<br>Convenience<br>n=549	Age not specified<br>Female	Yes	Blood sampling. Risk factors, demographics, and prevalence of high risk HPV assessed via structured interview.	HR HPV prevalence high and differed by geographic region of origin.
de Sanjose et al. (2002).<br>*Spain, Oveido & Barcelona.*<br>To determine prevalence of Kaposi Sarcoma associated Herpes in SWs compared to general population	Case control<br>Convenience<br>n=100 SWs, n=100 GP<br>RR= 50%	30.3 years<br>Female	Yes	Rates of HPV, HIV and KSHV collected by blood sampling	SWs twice as likely to be infected with KSHV than GP.

Folch et al. (2008).<br>*Spain, Catalonia.*<br>To determine prevalence of HIV, CT and NG according to region of origin and identify factors associated with current infection of CT and/or NG.	Cross-sectional<br>Convenience stratified<br>n=357	28 years<br>Female	Not specified	Oral fluid and urine samples for testing rates of HIV, CT and NG	Unprotected sex and younger age associated with CT and NG. Prevalence of STI in SWs lower in Catalonia than other European countries.
Gonzalez et al. (2011).<br>*Spain, Alicante.*<br>To compare incidence rates and persistence of HR-HPV infections in FSWs and general population	Cohort<br>Convenience<br>n=144 FSW, n=592 GP	29 years (SW), 34 years (GP)<br>Female	Yes	Cervical swab collection for testing rates of HPV.	FSWs had higher incidence and persistence of HR-HPV than WGP.
Pineda et al. (1992).<br>*Spain, Andalusia.*<br>To assess prevalence of and risk factors for HIV-1 among non-IV drug using SWs	Cross-sectional<br>Convenience<br>n=519<br>RR= 84%	30 years, Female	Yes	HIV testing. Structured interview to assess sexual practices and demographics.	Prevalence of HIV-1 low. Risk increased with higher rates of sexual exposure.
Pinedo Gonzales et al. (2018).<br>*Spain.*<br>To assess impacts of violence and drug use on loneliness	Cross-sectional<br>Purposive<br>n=146	30.88 years<br>Female, Transgender	Yes	Instances of violence, drug use and feelings of loneliness via questionnaire	Physical and psychological violence associated with increased loneliness levels. Increase in loneliness associated with drug use.
Rodríguez-Cerdeira et al. (2014).<br>*Spain, Ourense & Pontevedra.*<br>To determine socio-economic, lifestyle and behaviours of SWs	Cross-sectional<br>Convenience<br>n=168	28.2 years<br>Female	Yes	Demographics, socio-economics and sexual and healthcare practices via questionnaire.	Knowledge of pap smears moderate (47%). Poor awareness of HPV. 88% understood HIV transmitted sexually.

Table: S3. Summary of studies in 'Nordic model' jurisdictions (n=1)

**Study**<br>**Location**<br>**Aim**	**Study design**<br>**Recruitment**<br>**Sample (n)**<br>**Response Rate (RR)**	**Participant characteristics:**<br>**Mean age**<br>**Gender**	**Ethical approval**	**Evaluation design and measures**	**Study findings**
Benoit et al. (2016).<br>*Canada.*<br>To assess healthcare needs and reasons for not accessing healthcare	Cross-sectional<br>Purposive<br>n=209	34 years<br>Female, Male, Transgender	Yes	Perceived health care needs, access and health status via questionnaire	SWs had poor mental health and higher prevalence of unmet health care needs. SWs had greater dislike of doctors and fear of health services than the general population.

Table: S4. Summary of studies in legalised jurisdictions (n=18)

Study<br>Location<br>Aim	Study design<br>Recruitment<br>Sample (n)<br>Response Rate (RR)	Participant characteristics:<br>Mean age<br>Gender	Ethical approval	Evaluation design and measures	Study findings
Chen et al. (2010).<br>*Australia, Melbourne (VIC).*<br>To estimate number of unlicensed brothels and assess sexual health of women working in unlicensed brothels.	Cross-sectional<br>Convenience<br>n=22	Age not specified<br>Female	Yes	Tampon specimen collection for STI testing. Sexual health status via questionnaire.	Number of unlicensed brothels less than expected. SWs in unlicensed brothels had lower rates of STI testing than licensed brothels. Unregulated SW industry made it difficult to promote sexual health and STI control.
Chow et al. (2014).<br>*Australia, Melbourne (VIC).*<br>To evaluate the impact of reducing STI testing from monthly to 3 monthly	Cross-sectional<br>Convenience<br>n=6146 (monthly consults),<br>n=3453 (3 monthly consults)	Age not specified<br>Female, Male	Yes	Prevalence of STI diagnoses via data from clinic records	Less frequent screening did not result in an increase of STIs per 100 consultations or hours. Approximately a quarter of a million dollars redirected to other at-risk groups.
Groves et al. (2008).<br>*Australia, Victoria.*<br>To examine characteristics and work attitudes/approach of SWs	Cross-sectional<br>Convenience<br>n=97<br>RR=90%	No mean given, majority aged between 23-35 years<br>Female	Yes	Attitudes and behaviours of SWs via questionnaire	Women in sex industry had a range of backgrounds/circumstances and varying attitudes towards industry. High level of stigma associated with occupation.

Lee et al. (2005).<br>*Australia, Victoria.*<br>To determine incidence of STI among decriminalized and regulated SW	Clinical audit<br>Purposive<br>n=388	Age not specified<br>Female	Not required	Epidemiological data on demographics, STI prevalence and sexual health	STI incidence low amongst decriminalized and regulated SW.
Samaranayake et al. (2009).<br>*Australia, Victoria.*<br>To determine effects of changed legislation to monthly STI testing (from previous three monthly)	Cross-sectional<br>Purposive<br>n=5722 (consultations from medical records)	Age not specified<br>Female	Yes	Epidemiological risk profile, time of consultation and STI diagnoses via medical record data	Monthly STI testing resource intensive limiting access to testing. Monthly testing compromised access to high-risk groups.
Seib et al. (2009).<br>*Australia, Queensland.*<br>To assess variation in self-reported STI rates among private, and illegal SWs and licensed brothels	Cross-sectional<br>Convenience<br>n=247<br>RR=98% (brothels), RR=93% (street work)	32 years<br>Female	Yes	STI diagnoses via questionnaire	Lower STI rates reported by SWs from licensed brothels.
Seib et al. (2009).<br>*Australia, Queensland.*<br>To describe self-reported physical and mental health of SWs	Cross-sectional<br>Convenience<br>n=247<br>RR=98% (brothels), RR=93% (street work)	32 years, Female	Yes	Self-report data on status of physical and mental health via questionnaire	Illegal SWs, particularly street based SWs, were four times more likely to report poor mental health outcomes than SWs working in licensed brothels.

Tang et al. (2013).<br>*Australia, Melbourne (VIC).*<br>To determine prevalence of STIs from low prevalence and high prevalence countries	Cross-sectional<br>Convenience<br>n=4774	30.1 years, Female	Yes	Laboratory testing to determine rates of STIs. Sexual health service records including demographics.	Prevalence of STIs was low amongst SWs from low and high prevalence countries.
Prieto et al. (2018).<br>*Chile, Santiago.*<br>To determine socio-economic, epidemiological and behavioural characteristics of SWs who do/do not access sexual health services	Cross-sectional<br>Time-location<br>n=370	31 years<br>Female	Yes	Access to and use of health services via questionnaire	38.6% of participants had not accessed a sexual health check-up clinic. FSWs who did not get check-ups were more likely to be younger.
Resl et al. (2003).<br>*Czech Republic, West Bohemia Region.*<br>To determine prevalence of STIs	Cross-sectional<br>Convenience<br>n=561	Age not specified<br>Female	Not specified	STI screening	STI prevalence higher than general population. Half of participants were from bordering regions of Austria and Germany.
Moro et al. (2013).<br>*Hungary, Budapest.*<br>To assess prevalence and problems associated with drug use	Cross-sectional<br>Convenience<br>n=510	29.5 years<br>Female, Male	Not required	Demographic, alcohol and drug use data via questionnaire	SWs had higher lifetime prevalence of illicit drug use compared with general population.

Baars et al. (2009).<br>*The Netherlands.*<br>To explore reach of a free HBV vaccination program among FCSWs within a legalised SW setting in the Netherlands and investigate reasons for nonparticipation and noncompliance.	Cross-sectional<br>Purposive<br>n=259	33 years<br>Female	Not specified	Knowledge of sexual risk behaviours and vaccination awareness via structured interview	79% reported awareness of free HBV vaccination, 63% were vaccinated. Personal approach by health professionals associated with vaccination uptake. Window SW and duration of SW in the area associated with awareness.
Krumrei-Mancuso (2017)<br>*The Netherlands.*<br>To examine depression and PTSD in SWs in relation to characteristics of the job	Cross-sectional<br>Convenience<br>n=88	33.45 years<br>Female	Yes	Mental health assessment via quality of life questionnaire	Sense of fair treatment, achievement and self-acceptance associated with better mental health outcomes.
Marra et al. (2018).<br>*The Netherlands, Amsterdam.*<br>To determine prevalence and risk factors for HPV infection	Cross-sectional<br>Convenience<br>n=304<br>RR=51%	29 years<br>Female	Yes	HPV serology sampling	High HPV prevalence, indicating population at risk.
Van Veen et al. (2008).<br>*The Netherlands.*<br>To assess potential for HIV transmission in this population	Cross-sectional<br>Convenience<br>n=557	32 years<br>Female and Transgender	Yes	HIV saliva testing, Health knowledge of sexual risk behaviour via structured questionnaire.	HIV most prevalent amongst transgender SWs. 74% of participants who were HIV positive were unaware of HIV status. 81% reported consistent condom use.
Verscheijden et al. (2015).<br>*The Netherlands.*<br>To investigate rate of STIs and determinants of diagnosis	Cross-sectional<br>Purposive<br>n=36,164 (consultations)	30 years<br>Female	Not required	Demographics, STI diagnosis, sexual behaviour via STI clinic data	Stable STI positivity rate. Decreasing chlamydia diagnoses and increasing, gonorrhoea diagnoses.

Hakre et al. (2013).<br>*Panama.*<br>To investigate prevalence of HIV, STIs and risk factors<br>*Legal= Sex work*<br>*Illegal= Procuring sex work*	Cross-sectional<br>Time-location<br>n=1000<br>RR=80%	29.4 years<br>Female	Yes	STI testing. Risk behaviours via anonymous structured questionnaire	HIV prevalence low amongst SWs. Unregistered SWs had higher rates of HIV and STIs than registered SWs.
Rossler et al. (2010).<br>*Switzerland, Zurich.*<br>To determine mental health status of FSW in different settings/nationalities	Cross-sectional<br>Non-proportional quota<br>n=193	32.1 years<br>Female	Yes	Mental health risk factors and rates of violence via self-report questionnaire	High rates of mental health disorders. SWs exposed to high levels of violence.

Table: S5. Summary of studies in decriminalized jurisdictions (n=5)

Study<br>Location<br>Aim	Study design<br>Recruitment<br>Sample (n)<br>Response Rate (RR)	Participant characteristics:<br>Mean age<br>Gender	Ethical approval	Evaluation design and measures	Study findings
Abel (2014).<br>*New Zealand.*<br>To assess utilisation of health services post decriminalization	Cross-sectional<br>Purposive<br>n=772	Age not specified<br>Female, Male, Transgender	Yes	Self-reported use of health services by questionnaire	Majority of SWs completed regular health checks. Failure to report occupation due to stigma affected comprehensiveness of health checks for SWs.
Foster, McCormack, Thng, Wand and McNulty (2018).<br>*Australia, Sydney (NSW).*<br>To determine factors associated with condom use	Cross-sectional<br>Convenience<br>n=435<br>RR=89%	31 years<br>Female	Yes	Condom usage via self-report questionnaire	72% reported consistent condom use. Decrease in condom use found since similar study completed in 2003.
O'Connor, Berry, Rohrsheim and Donovan (1996).<br>*Australia, Sydney (NSW).*<br>To compare sexual health and predictors of condom usage between local and international SW	Cross-sectional<br>Purposive<br>n=214	25.5 years (local SW),<br>25.3 years (international SW)<br>Female	Yes	Demographics and behavioural and morbidity data via medical records	International SWs demonstrated higher rates of STIs and inconsistent condom use than local SWs. Reduced STI prevalence compared to data of 10 years prior.

Pell, Dabbhadatta, Harcourt, Tribe and O'Connor (2006).<br>*Australia, Sydney (NSW).*<br>To compare workplace conditions, demographics and sexual health of Asian SWs	Cross-sectional<br>Convenience<br>n=91 (1993), n=165 (2003)	26 years (1993)<br>33 years (2003)<br>Female	Yes	Condom usage, sex work environment and health service use via questionnaire	Condom usage, work conditions and access to health services improved from 1993 to 2003. Alcohol and drug usage was low.
Read, Wand, Guy, Donovan and McNulty (2012).<br>*Australia, Sydney (NSW).*<br>To determine predictors of inconsistent condom use for fellatio and prevalence of pharyngeal gonorrhoea	Cross-sectional<br>Convenience<br>n=1636	30 years<br>Female	Yes	Demographics and STI diagnosis data via sexual health service records	Chinese speaking SWs reported inconsistent condom use. High usage of condom for fellatio. Condom use varied significantly across age/language groups.

Table: S6. Summary of studies involving multiple jurisdictions with different regulatory approaches (n=5)

Study<br>Location<br>Aim	Study design<br>Recruitment<br>Sample (n)<br>Response Rate (RR)	Participant characteristics:<br>Mean age<br>Gender	Ethical approval	Evaluation design and measures	Study findings
Harcourt et al. (2010).<br>*Australia, NSW, VIC, WA*<br>To assess whether law impacted on delivery of health promotion programs	Cross-sectional<br>Convenience<br>n=605	Age not specified<br>Female	Yes	Characteristics of SW in relation to law via questionnaire	Legal context appeared to affect conduct of health promotion programs. Brothel licensing and police-controlled illegal brothels resulted in isolation from peer education and support in unlicensed brothels.
Li et al. (2016).<br>*Australia, Adelaide (SA), Melbourne (VIC) & Sydney (NSW).*<br>To compare gonorrhoea diagnoses in Adelaide with two Australian capital cities of differing legislation	Prevalence<br>Purposive<br>n=not specified	Age not specified<br>Female	Yes	Assessment of sexual health service records including demographic, clinical and epidemiological data.	Proportion of gonorrhoea diagnoses for Adelaide SWs was higher than data from Melbourne for same time period. Sydney SWs had significantly less gonorrhoea diagnoses.
Minichiello et al. (2000).<br>*Australia, Sydney (NSW), Melbourne (VIC) & Brisbane (QLD).*<br>To determine characteristics of MSW	Cross-sectional<br>Convenience<br>n=186<br>RR=50-95%	27.06 years<br>Male	Yes	Characteristics of MSW via self-complete diary	Street based MSW more likely to report unsafe sex practices.

Minichiello et al. (2001).<br>*Australia, Sydney (NSW), Melbourne (VIC) & Brisbane (QLD).*<br>To investigate socio-demographics and characteristics of SW	Cross-sectional<br>Convenience<br>n=185	26.98 years<br>Male	Yes	Law impact on SW and socio-demographic information via questionnaire	Legal context affected where SWs operated and varied by city. Sydney had fewer street-based SWs. A growing number of MSW chose SW as a career and considered it a profession.
Minichiello et al. (2003).<br>*Australia, Sydney (NSW) Melbourne (VIC) and Brisbane (QLD).*<br>To examine self-reported patterns of drug and alcohol use	Cross-sectional<br>Convenience<br>n=186	27 years<br>Male	Yes	Characteristics of drug and alcohol use via self-complete diary	AOD use moderated by contextual influences such as place, time and duration of client encounter. Drug use associated with increased likelihood of unsafe sex behaviours.
